# Evaluating a Web-Based Social Anxiety Intervention Among Community Users: Analysis of Real-World Data

**DOI:** 10.2196/11566

**Published:** 2019-01-10

**Authors:** Hugh Cameron McCall, Fjola Dogg Helgadottir, Ross G Menzies, Heather D Hadjistavropoulos, Frances S Chen

**Affiliations:** 1 Department of Psychology University of Regina Regina, SK Canada; 2 AI-Therapy North Vancouver, BC Canada; 3 Dr. Fjóla & Kompaní Reykjavík Iceland; 4 Hugræna Atferlisstöðin í Reykjavík Reykjavík Iceland; 5 Graduate School of Health University of Technology Sydney Sydney Australia; 6 Department of Psychology University of British Columbia Vancouver, BC Canada

**Keywords:** social anxiety, internet, cognitive behavioral therapy, psychotherapy, mental health

## Abstract

**Background:**

Social anxiety is both harmful and prevalent. It also currently remains among the most undertreated major mental disorders, due, in part, to socially anxious individuals’ concerns about the stigma and expense of seeking help. The privacy and affordability of computer-aided psychotherapy interventions may render them particularly helpful in addressing these concerns, and they are also highly scalable, but most tend to be only somewhat effective without therapist support. However, a recent evaluation of a new self-guided, 7-module internet-delivered cognitive behavioral therapy intervention called Overcome Social Anxiety found that it was highly effective.

**Objective:**

The initial evaluation of Overcome Social Anxiety revealed that it led to significant reductions in symptom severity among university undergraduates. The aim of this study was to extend the results of the initial study and investigate their generalizability by directly evaluating the intervention’s effectiveness among a general community sample.

**Methods:**

While signing up for Overcome Social Anxiety, users consented to the usage of their anonymized outcome data for research purposes. Before and after completing the intervention, users completed the Fear of Negative Evaluation Scale (FNE), which we employed as the primary outcome measure. Secondary outcome measures included the Depression Anxiety Stress Scales (DASS) and 2 bespoke questionnaires measuring socially anxious thoughts (Thoughts Questionnaire) and avoidance behaviors (Avoidance Questionnaire).

**Results:**

Participants who completed the intervention (102/369, 27.7%) experienced significant reductions in the severity of their symptoms on all measures employed, including FNE (*P*<.001; Cohen *d*=1.76), the depression subscale of DASS (*P*<.001; Cohen *d*=0.70), the anxiety subscale of DASS (*P*<.001; Cohen *d*=0.74), the stress subscale of DASS (*P*<.001; Cohen *d*=0.80), the Thoughts Questionnaire (*P*<.001; Cohen *d*=1.46), and the Avoidance Questionnaire (*P*<.001; Cohen *d*=1.42).

**Conclusions:**

Our results provide further evidence that Overcome Social Anxiety reduces the severity of social anxiety symptoms among those who complete it and suggest that its effectiveness extends to the general community. The completion rate is the highest documented for a fully automated intervention for anxiety, depression, or low mood in a real community sample. In addition, our results indicate that Overcome Social Anxiety reduces the severity of symptoms of depression, physiological symptoms of anxiety, and stress in addition to symptoms of social anxiety.

## Introduction

### Background

Social anxiety disorder has a high lifetime prevalence of approximately 13% [1]. It causes considerable distress and functional impairment, even at a subclinical level of severity [2], and impacts both the private and professional lives of those affected by it [3,4]. It is persistent in the absence of treatment [5] and is related to other mental disorders such as mood and substance disorders [6]. Yet, social anxiety remains one of the most undertreated of all major mental disorders today [7]. Importantly, its relatively low treatment rate cannot be attributed to any lack of empirically supported treatment methods; research has shown that both psychotherapeutic treatments (eg, cognitive behavioral therapy, CBT) and pharmaceutical treatments (eg, selective serotonin reuptake inhibitors) for social anxiety are effective [8,9]. Rather, financial constraints and concerns about being judged or stigmatized for seeking help, among other issues, represent major barriers to treatment for socially anxious individuals [7].

Computerized cognitive behavioral therapy (CCBT), a promising and increasingly popular treatment for anxiety and depression [10], may be particularly useful in surmounting social anxiety’s unique barriers to treatment, as it is more affordable and can be accessed more privately than traditional psychotherapy. Most computer-aided psychotherapy (CP) interventions (a category of interventions including CCBT) involve some therapist support, and the effectiveness of CP interventions is related to the amount of therapist support their users receive [11,12]. Accordingly, self-guided CCBT interventions—those designed to operate independently, without the necessity of therapist support—tend to be less effective than those involving therapist support [13].

### Overcome Social Anxiety

A recent study suggests that a self-guided internet-delivered cognitive behavioral therapy (ICBT; CCBT delivered via the internet) intervention called Overcome Social Anxiety may represent a notable exception to the tendency for self-guided CCBT interventions to be less effective than those involving therapist support [14]. The study—a randomized controlled trial among undergraduate university students, which compared Overcome Social Anxiety with a wait-list control condition—revealed a between-groups effect size (Cohen *d*=0.97) similar to the average between-groups effect size of 19 trials of computer-aided interventions *with* therapist support found in a review (Cohen *d*=0.96) [11].

Overcome Social Anxiety arose from a program of research exploring the common limitations of other CCBT interventions [15] and was designed to address 5 such limitations in particular. First, where many other interventions do not adequately individualize treatment to individual participants’ needs, Overcome Social Anxiety employs a series of questionnaires to tailor each user’s treatment package to address their unique symptoms and the contexts in which those symptoms typically occur. Second, corrective feedback on important aspects of the CBT process is often lacking from CCBT interventions; Overcome Social Anxiety mitigates the need for corrective feedback by providing example responses to help ensure that users fully understand what they are required to do at each stage of the treatment process (eg, challenging maladaptive thoughts and designing behavioral experiments). Third, where some interventions do not adequately address low adherence rates [15], which remain common among CP interventions today [16], Overcome Social Anxiety employs 2 mechanisms to encourage users to make steady progress: (1) it hinders procrastination by limiting users to a 6-month window to complete the intervention and (2) it mitigates forgetfulness by sending users automated reminders to continue their work on the program following periods of inactivity. Fourth, although research shows that therapist-client interaction is an important aspect of successful CBT [17], it is by definition lacking from stand-alone CCBT interventions. Overcome Social Anxiety addresses this challenge by employing voice recordings of 2 clinical psychologists to guide users through the treatment process, more closely mirroring a traditional course of human-delivered therapy. A recent study found that the *patient-program alliance*, similar to the therapeutic alliance, is associated with greater adherence and more favorable clinical outcomes in CCBT [18], indicating that such efforts at improving how users relate to interventions themselves are unlikely to be misguided. Finally, many other interventions have failed to provide users with a sufficient dose of treatment to effect lasting positive change [15], despite research attesting to the importance of an appropriate dose of treatment to CBT’s success [19]. Overcome Social Anxiety was designed to deliver a more robust treatment package than many other programs and includes all established elements of modern CBT.

Overcome Social Anxiety comprises an assessment battery and 7 core modules. The intervention begins with a *prequestionnaires* module, which is designed both to take a pretreatment measure of users’ symptom severity and to individualize the treatment to each user. Module 1, *Thinking exercises*, introduces users to the program and their virtual psychologists, informs users of common cognitive errors, and explains the relationship between cognitions, behaviors, and emotions. Module 2, *Challenging your thinking*, presents users with personally relevant anxious thoughts (based on prequestionnaire responses) and asks users to challenge those thoughts through writing exercises. In module 3, *Creating your model*, users select symptoms and anxiety-inducing situations and cognitions, which the intervention then uses to individualize the treatment to users’ unique experiences of social anxiety. This model is then applied to module 4, *Behavioral experiments*, wherein users are guided through a series of behavioral experiments to target safety behaviors and avoidance. Module 5, *Challenge your thinking further*, continues to help users adjust their negative beliefs, with a particular emphasis on anger. Module 6, *Self-processing*, targets biased attentional processes through skills-based attention training [20] and rescripting of faulty and negative imagery [21]. Module 7, *Relapse prevention*, reviews the material covered in the first 6 core modules and provides users with psychoeducation to help them maintain treatment gains. Finally, users complete the *postquestionnaires* module, which the program uses to create histograms to show users the difference between their pre- and posttreatment symptom severity. The program then provides users with an individualized PDF containing all program materials, which users can employ to help maintain treatment gains into the future.

Each module was designed to achieve a particular clinical goal and not necessarily to be completed in a single sitting. Indeed, some require substantially more time and effort than others. For example, when users are taught how to change their thinking in module 2, they are encouraged to target 1 thought they are working on changing per day. Please see the initial trial [14] for more detailed information about the content of the program and Multimedia Appendix 1 for a screenshot.

The results of the initial trial indicate that the 5 design features discussed above may be collectively very useful, potentially contributing enough to bridge the effectiveness gap between self-guided and therapist-assisted interventions. Overcome Social Anxiety—if it is indeed as effective as the initial trial suggests—may have important implications not only for reducing the severity of symptoms and increasing the well-being of people who struggle with social anxiety but also for the development of future interventions.

Although the initial study was a randomized controlled trial with high internal validity, it did not explicitly investigate the generalizability of its findings to the general community of individuals with social anxiety. The purpose of this study was to attempt to replicate the findings of the initial trial with high external validity by employing Overcome Social Anxiety’s general user base as its sample. Our rationale for conducting this study was that—to the extent that the intervention was found to be similarly effective among its general user base as it was among the initial trial’s student sample—consumers, mental health professionals, researchers, and developers of future ICBT interventions would be able to place more confidence in the intervention’s effectiveness.

### Hypothesis

We hypothesized that among those who completed all modules of the intervention (including its prequestionnaires module, its 7 core modules, and its postquestionnaires module), referred to throughout this study as *completers*, posttreatment scores on the Fear of Negative Evaluation Scale (FNE) [22] would be significantly lower than pretreatment scores. The results from the initial trial [14], which also employed FNE and found a large pretreatment-to-posttreatment effect size for that measure (Cohen *d*=0.82), suggested that this difference could be large.

## Methods

### Participants

In this study, we retrospectively analyzed data from past users of Overcome Social Anxiety. These data were automatically collected from users between August 2012 and April 2018. Thus, no new data collection was necessary. Our sample (n=369) consisted of all former, paying users of Overcome Social Anxiety. We excluded (1) those whose usage of the program was ongoing as of April 2018, (2) the university undergraduates who participated in the initial trial [14], and (3) all other users who were given free access to the intervention (eg, through the private practices of its creators).

A power analysis suggested that, for within-subjects comparisons of pre- and posttreatment FNE scores, a sample of 40 completers would have been required to achieve a power level of 0.99 at the *P*<.01 level of significance, two-tailed, assuming the effect size of Cohen *d*=0.82 found in the initial trial [14]. Even given a conservative estimate of an effect half this size (ie, Cohen *d*=0.41) for this study’s population, we would have achieved a power level of 0.93 at the *P*<.01 level of significance, two-tailed, with the 102 users who completed the intervention.

All past and present users of Overcome Social Anxiety consented to the collection, anonymization, and later analysis of their data for research purposes during registration. The protocol for this study was approved by the University of British Columbia’s Behavioural Research Ethics Board (Human Ethics Application ID: H16-00319).

### Outcome Measures

Overcome Social Anxiety begins with a prequestionnaires module, which is included both to individualize the course of treatment to each user’s needs and to measure each user’s pretreatment symptom severity. The intervention concludes with a postquestionnaires module, which allows users to quantify changes in the severity of their symptoms. Both these modules contain 4 measures. First, they contain the FNE scale [22], a well-validated measure of social anxiety symptoms [22,23]. The brief FNE scale, whose scores have been found to share a Pearson correlation coefficient of .96 with the original FNE (*r*=.96) [24], correlates with other measures of social anxiety and yields significantly higher scores among those diagnosed with social anxiety disorder than those without [25]. The FNE comprises 30 statements (eg, “I worry that others will think I am not worthwhile”) that respondents mark as true or false and yields a total score between 0 and 30. Scores of 7 (1 SD below the mean for a large student sample) and 8 (lower quartile) have been recommended as cut-off scores to indicate low social anxiety, whereas scores of 22 (1 SD above the mean) and 20 (upper quartile) have been recommended as cut-off scores to indicate high social anxiety [23].

Second, both these modules include the Depression Anxiety Stress Scales (DASS) [26], a 42-item questionnaire designed to discriminate between depression (eg, “I felt sad and depressed”), anxiety (eg, “I was aware of dryness of my mouth”), and stress (eg, “I was in a state of nervous tension”), despite their shared symptoms. Research has attested to its reliability and validity [27,28].

Finally, these modules include 2 bespoke questionnaires, which ask users about the frequency with which they experience 37 socially anxious thoughts (eg, “I can’t speak to authority figures”) and avoid 23 anxiety-provoking situations (eg, “Making small talk with strangers/colleagues”). The items on these 2 questionnaires—titled the Thoughts Questionnaire and the Avoidance Questionnaire, respectively—were retrieved from a file audit of decades of clinical psychology practice with individuals diagnosed with social anxiety. Both questionnaires are scored on 5-point Likert scales and are intended to capture the patterns of thinking and behavior characteristic of real experience with social anxiety symptoms. Cronbach alphas for the Thoughts Questionnaire and the Avoidance Questionnaire were .94 and .89, respectively. In our sample, participants’ scores on FNE were related to their scores on both bespoke questionnaires, with Pearson correlation coefficients of .58 (n=369; *P*<.001) for the Thoughts Questionnaire and .36 (n=369; *P*<.001) for the Avoidance Questionnaire.

### Procedure

**Figure 1:**
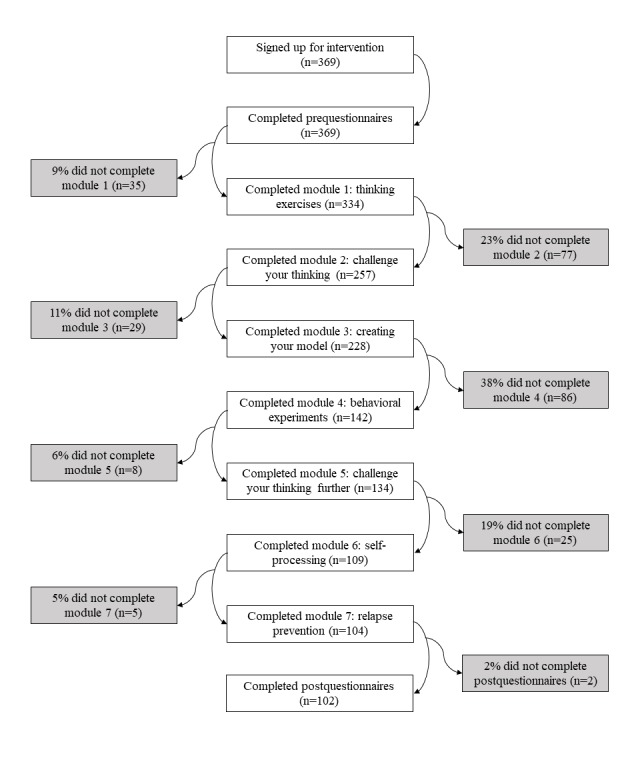
Flowchart of user progress.

Users from around the world found Overcome Social Anxiety independently and chose to sign up for US $149.99. The intervention was advertised through Google Adwords between August 2012 and August 2014, has received media coverage, and has seen mention in blog posts, which likely helped users to discover it. During registration, users consented to the use of their data for research. After signing up, users first completed the prequestionnaires module, consisting of FNE, DASS, the Thoughts Questionnaire, and the Avoidance Questionnaire. They then began working through the 7 core modules of the program (see Figure 1 for content outline and the initial trial [14] for details). On completing all the program’s core modules, users responded to FNE, DASS, the Thoughts Questionnaire, and the Avoidance Questionnaire again during the postquestionnaires module. At the end of this module, they were also asked to leave feedback for the creators of the intervention. Users were given a limit of 6 months from the date of their registration to complete the intervention. They were sent automated emails reminding them to continue using the program after 3, 7, 10, 14, 21, and 28 days of inactivity. On completing each module, users were also sent automated emails summarizing that module’s contents. As this study involved only the retrospective analysis of data from past users of Overcome Social Anxiety, there was no contact with participants throughout the course of the study.

### Analyses

This study’s primary dependent variable was pretreatment-to-posttreatment change in the severity of social anxiety symptoms, as measured by FNE. We selected FNE as a primary measure because it is a well-established and validated measure of social anxiety symptoms and because our other measure of anxiety, the anxiety subscale of DASS, measures primarily physiological symptoms of anxiety and does not specifically measure social anxiety symptoms. However, we also analyzed changes in scores on 5 secondary measures—the 3 factors of DASS, the Thoughts Questionnaire, and the Avoidance Questionnaire. For each of these measures, within-subjects *t* tests were conducted to determine whether posttreatment scores differed from pretreatment scores among users who completed the intervention.

We tested for attrition bias by exploring differences between completers and noncompleters. Specifically, we conducted between-subjects *t* tests to check for differences between completers and noncompleters in age and pretreatment scores on all questionnaire measures. We also conducted Chi-square analyses for sex and whether users reported having previously seen a therapist for anxiety, seen a therapist for other reasons, or taken medication for anxiety. Finally, after imputing missing posttreatment data from noncompleters using a last observation carried forward approach, we conducted between-subjects *t* tests to check for pretreatment-to-posttreatment changes in symptoms on all outcome measures among all users, completers and noncompleters alike.

At the end of the postquestionnaires module, completers were asked to leave positive and negative feedback about the intervention. Employing a conventional content analysis approach [29] and QSR NVivo 11 software, we used both qualitative methods (coding participants’ responses and grouping them into themes) and quantitative methods (counting comments reflecting each theme and calculating descriptive statistics) to analyze participants’ responses [30]. Two researchers (KG—see Acknowledgments below—and author HM) carefully read each comment and collaboratively created a coding guide. KG used this guide to code each response and then met HM again to discuss potential inconsistencies in the application of the coding guide to the analysis of participants’ comments. Finally, an expert coder (author HH) conducted a final review of the coded comments, counted comments reflecting each theme, and calculated descriptive statistics.

## Results

### Program Usage

Of the 369 users who signed up for Overcome Social Anxiety between August 2012 and April 2018, 102 users (102/369, 27.7%) fully completed the intervention. The number of users who completed each module of the intervention is displayed in Figure 1. The average time taken for completion of each module, in minutes, was 14.70 for the prequestionnaires, 34.60 for module 1, 83.70 for module 2, 43.10 for module 3, 103.72 for module 4, 15.02 for module 5, 25.05 for module 6, 8.05 for module 7, and 19.10 for the postquestionnaires. Completers spent a mean of 5 hours and 34 min (SD 211 min) using the intervention, whereas noncompleters spent a mean of 2 hours and 11 min (SD 86 min). However, these data represent only the amount of time users spent logged in to the intervention. Important components of CBT, including homework exercises such as exposure activities, occur between sessions, and the overall amount of time spent is likely to be considerably higher. On average, completers logged in 25.06 times (SD 17.25) over a period of 138.07 days (SD 126.07), whereas noncompleters logged in 9.93 times (SD 8.14) over a period of 58.02 days (SD 83.27).

### User Characteristics

Out of the 102 completers, 39 (38.2%) identified as female, 58 (56.9%) identified as male, and 5 (4.9%) did not report their sex. The mean age of completers was 35.47 (SD 13.64). When asked about their clinical history, many completers reported having previously seen a therapist for anxiety (45/94, 48%), seen a therapist for another reason (43/95, 45%), and taken medication for anxiety (36/96, 38%). These data, in addition to those of noncompleters, are displayed in Table 1. The most common countries of residence users reported during registration were the United States (105/369, 28.5%), the United Kingdom (56/369, 15.2%), Australia (55/369, 15.0%), Iceland (30/369, 8.1%), and Canada (28/369, 7.6%). The remaining users were spread across 25 other countries around the world (60/369, 16.3%) or did not report their countries of residence (35/369, 9.5%).

### Pretreatment Questionnaire Scores

The mean pretreatment FNE score among completers was 25.91 (SD 3.99), indicating very high levels of social anxiety. This score approached the FNE’s maximum score of 30 and exceeded cut-off scores defining high anxiety (20 and 22) by a considerable margin [23]. For DASS, completers had pretreatment scores of 14.07 (SD 10.07) on the depression subscale, 9.93 (SD 6.84) on the anxiety subscale, and 16.28 (SD 8.05) on the stress subscale. Finally, completers’ mean pretreatment scores on the Thoughts Questionnaire and Avoidance Questionnaire were 79.72 (SD 23.4) and 51.37 (SD 14.85), respectively.

### Comparison of Completers and Noncompleters

A between-subjects *t* test revealed that completers had lower scores than noncompleters on the anxiety subscale of DASS (equal variances not assumed; *t*_217.66_=2.95; *P*=.003; Cohen *d*=−0.33). Between-subjects *t* tests of DASS’s depression and stress subscales, FNE, the Thoughts Questionnaire, the Avoidance Questionnaire, and age revealed no further differences between completers and noncompleters (all *P* s>.07). In addition, Chi-square analyses revealed no differences between completers and noncompleters in sex ratio or whether users reported having previously seen therapists for anxiety, seen therapists for other reasons, or taken medication for anxiety (all *P* values> .15).

### Effectiveness of the Intervention

Completers experienced significant pretreatment-to-posttreatment reductions in symptom severity on all measures employed: FNE (*t*_101_=13.61; *P*<.001; Cohen *d*=1.76), the depression subscale of DASS (*t*_101_=7.42; *P*<.001; Cohen *d*=0.70), the anxiety subscale of DASS (*t*_101_=8.24; *P*<.001; Cohen *d*=0.74), the stress subscale of DASS (*t*_101_=9.57; *P*<.001; Cohen *d*=0.80), the Thoughts Questionnaire (*t*_101_=16.47; *P*<.001; Cohen *d*=1.46), and the Avoidance Questionnaire (*t*_101_=15.40; *P*<.001; Cohen *d*=1.42). Demographic characteristics and pretreatment questionnaire scores for all users, in addition to posttreatment questionnaire scores and symptom change analyses for completers, are summarized in Table 1.

For each outcome measure, some completers reported a worsening in symptom severity from pre- to posttreatment. These changes were all less than 1 SD (ie, 1 pretreatment SD among completers for each measure) in magnitude, except for those of 3 (3/102, 3.0%) participants for FNE, 4 (4/102, 4.0%) for the depression subscale of DASS, 1 (1/102, 1.0%) for the anxiety subscale of DASS, and 1 (1/102, 1.0%) for the stress subscale of DASS.

As discussed above in outcome measures, FNE threshold scores of 20 and 22 have been recommended to distinguish between high anxiety and moderate or low anxiety individuals [23]. According to these thresholds, 96 (96/102, 94.1%) or 90 (90/102, 88.2%) completers reported high anxiety before beginning the clinical content of the intervention, whereas 33 (33/102, 32.4%) or 28 (28/102, 27.5%) reported high anxiety after completing it. One completer began the intervention below 1 of the thresholds (with a score of 20) and ended it above that threshold (with a score of 24). All other participants either remained in the same category of anxiety or experienced a reduction in their FNE score, which moved them past a threshold and into a lower category of anxiety.

**Table 1:** User characteristics and questionnaire scores.

Characteristic			Completers (n=102)	Noncompleters (n=267)	Total (N=369)
**User characteristics^a^**					
	Female, n (%)		39 (40.2)	98 (38.4)	137 (39.0)
	Age, mean (SD)^b^		35.47 (13.64)	33.88 (11.93)	34.31 (12.42)
	Seen therapist for anxiety, n (%)		45 (47.9)	129 (52.0)	174 (50.9)
	Seen therapist for other reasons, n (%)		43 (45.3)	96 (38.9)	139 (40.6)
	Taken medication for anxiety, n (%)		36 (37.5)	120 (48.6)	156 (45.5)
**Fear of negative evaluation**					
	Pretreatment, mean (SD)		25.91 (3.99)	25.53 (5.05)	25.63 (4.78)
	Posttreatment, mean (SD)		15.06 (8.32)	—	—
	**Change^c^**				
		*t* test (*df*)	13.61 (101)	—	—
		*P* value	<.001	—	—
		Cohen *d*	1.76	—	—
**DASS^d^** **(depression subscale)**					
	Pretreatment, mean (SD)		14.07 (10.07)	16.34 (11.28)	15.72 (10.99)
	Posttreatment, mean (SD)		7.57 (8.60)	—	—
	**Change^c^**				
		*t* test (*df*)	7.42 (101)	—	—
		*P* value	<.001	—	—
		Cohen *d*	0.70	—	—
**DASS (anxiety subscale)**					
	Pretreatment, mean (SD)		9.93 (6.84)	12.42 (8.212)	11.73 (7.93)
	Posttreatment, mean (SD)		5.32 (5.64)	—	—
	**Change^c^**				
		*t* test (*df*)	8.24 (101)	—	—
		*P* value	<.001	—	—
		Cohen *d*	0.74	—	—
**DASS (stress subscale)**					
	Pretreatment, mean (SD)		16.28 (8.05)	18.14 (9.40)	17.63 (9.08)
	Posttreatment, mean (SD)		10.01 (7.63)	—	—
	**Change^c^**				
		*t* test (*df*)	9.57 (101)	—	—
		*P* value	<.001	—	—
		Cohen *d*	0.80	—	—
**Thoughts Questionnaire**					
	Pretreatment, mean (SD)		79.72 (23.40)	80.66 (23.68)	80.40 (23.57)
	Posttreatment, mean (SD)		44.93 (24.17)	—	—
	**Change^c^**				
		*t* test (*df*)	16.47 (101)	—	—
		*P* value	<.001	—	—
		Cohen *d*	1.46	—	—
**Avoidance Questionnaire**					
	Pretreatment, mean (SD)		51.37 (14.85)	52.35 (15.59)	52.08 (15.37)
	Posttreatment, mean (SD)		29.54 (15.97)	—	—
	**Change^c^**				
		*t* test (*df*)	15.40 (101)	—	—
		*P* value	<.001	—	—
		Cohen *d*	1.42	—	—

^a^Some users did not respond to certain questions. The 5 rows beneath this heading display responses from 97 completers and 255 noncompleters who reported their sex, 92 completers and 246 noncompleters who reported their age, 94 completers and 248 noncompleters who reported whether or not they had previously seen a therapist for anxiety, 95 completers and 247 noncompleters who reported whether or not they had previously seen a therapist for another reason, and 96 completers and 247 noncompleters who reported whether or not they had previously taken medication for anxiety. The percentages given represent percentages of respondents, not percentages of participants overall.

^b^Age was measured by year of birth, and our statistics on users’ ages represent their ages at the end of the calendar years during which they began the intervention.

^c^This row displays the results of a within-subjects *t* test comparing completers’ pre- and posttreatment scores.

^d^DASS: Depression Anxiety Stress Scales.

### Imputation of Missing Data

It has been suggested that where dropout rates exceed 20%—and this study’s dropout rate (267/369, 72.4%) did so by far—“no adequate recommendation [for replacing missing data] can be provided” [31]. However, research has demonstrated that partial completion of ICBT interventions for anxiety and depression leads to symptom reduction [32], and noncompleters in our sample spent an average of over 2 hours using Overcome Social Anxiety, suggesting that noncompleters may have benefited from the intervention. For this reason, imputation of missing data from noncompleters using a last observation carried forward approach may be conservative. A within-subjects *t* test comparing pre- and posttreatment FNE scores for all users, assuming no pretreatment-to-posttreatment change in FNE scores for noncompleters, indicated that the program was moderately effective in reducing symptoms among all users (*t*_368_=8.95; *P*<.001; Cohen *d*=0.48). Even in the hypothetical and unlikely event that noncompleters experienced an increase in social anxiety symptoms equivalent to half an SD on FNE (ie, a score increase of 2.52; Cohen *d*=0.5), our results would show a small but statistically significant reduction in FNE scores among all users (*t*_368_=3.08; *P*=.002; Cohen *d*=0.18).

Furthermore, within-subjects *t* tests comparing pre- and posttreatment scores on secondary outcome measures—again, assuming no change in score for noncompleters from pre- to posttreatment—showed significant, small-to-moderate reductions in symptom severity on all secondary outcome measures: the depression subscale of DASS (*t*_368_=6.31; *P*<.001; Cohen *d*=0.16), the anxiety subscale of DASS (*t*_368_=6.78; *P*<.001; Cohen *d*=0.16), the stress subscale of DASS (*t*_368_=7.46; *P*<.001; Cohen *d*=0.19), the Thoughts Questionnaire (*t*_368_=9.63; *P*<.001; Cohen *d*=0.37), and the Avoidance Questionnaire (*t*_368_=9.41; *P*<.001; Cohen *d*=0.35).

### Acceptability of the Intervention

Analysis of user feedback identified 9 positive themes and 8 areas for improvement. These themes, the number of participants whose comments reflected them, and examples of these comments are displayed in Table 2. It is worth noting that feedback was only obtained from users on their completion of the program and that users were asked about both what they liked and what they did not like about the intervention. Out of 102 completers, 35 users left comments. The mean number of comments coded as positive feedback was 1.77 (SD 1.17), whereas the mean number of comments coded as areas for improvement was 0.80 (SD 0.83).

**Table 2:** Feedback from completers.

Feedback theme		n (%)
**Positive feedback**		
	General praise (eg, *I loved this program.*)	26 (25.5)
	Specific symptom improvement (eg, *...this program truly did help me overcome a lot of the thoughts I was having.*)	18 (17.6)
	Content quality (eg, *...shows deep understanding of the problems of social anxiety.*)	12 (11.8)
	Components (eg, *...I find the e book invaluable...*)	9 (8.8)
	Presentation (eg, *Liked A variety of pictures, sound and interaction.*)	9 (8.8)
	Convenience or accessibility (eg, *Good to be able to do things completely at your own speed.*)	6 (5.9)
	Cost (eg, *Not too expensive.*)	3 (2.9)
	Privacy (eg, *AI Therapy’s anonymous and confidential form of treatment has been wonderful...*)	3 (2.9)
	Ease of use (eg, *Easy to use. Great format. Very user friendly.*)	2 (2.0)
**Areas for improvement**		
	Components (eg, *I found some of the content helpful, but there were some bits that I didn’t find help nor did it relate to me.*)	10 (9.8)
	Presentation (eg, *...I wish it was a little more visually interesting.*)	7 (6.9)
	Content quality (eg, *Would be good for anxious teenagers, but somewhat too simple for adults.*)	4 (3.9)
	Research considerations (eg, *...questionnaires were a bit long.*)	2 (2.0)
	Technical problems (eg, *Little bit glitchy on an iPad.*)	2 (2.0)
	Cost (eg, *Not much more help provided than self-help books which cost a lot less.*)	1 (1.0)
	Specific lack of symptom improvement (eg, *I liked it even though i dont feel much improvement in my overall case...*)	1 (1.0)
	Length (eg, *...it was a little ’longer’ than I expected due to having to go out and face our fears...*)	1 (1.0)

## Discussion

### Principal Findings

Our primary hypothesis was that those who completed the intervention would experience a significant reduction in the severity of their social anxiety symptoms, as measured by FNE [22]. This hypothesis was clearly supported by our results. In fact, the effect size for this reduction (Cohen *d*=1.76) was larger than the mean uncontrolled pretreatment-to-posttreatment effect size of *human-delivered* CBT for social anxiety found in a meta-analysis (effect size=1.04) [33]. By comparison, although this study lacked a control condition, participants in the waitlist control condition of our initial trial experienced a mean reduction in FNE scores of 0.46 (Cohen *d*=0.14) over a similar length of time [14]. Social anxiety is widely considered to have a chronic course, and clinical and epidemiological studies have reported that it has a mean duration of 10 to 24 years [34]. Given this typical time course of social anxiety, in combination with the results for the waitlist control condition of our initial trial, we infer that the reduction in severity of social anxiety symptoms in this study is attributable primarily to the intervention rather than to spontaneous remission.

Our results also show that those who completed the intervention experienced reductions not only in the severity of social anxiety symptoms, as measured by FNE, but also in the severity of symptoms of depression, physiological symptoms of anxiety, and stress, as measured by DASS, and self-reported socially anxious thoughts and avoidance behaviors, as measured by the Thoughts Questionnaire and the Avoidance Questionnaire. Given that social anxiety is related to depression [35], general anxiety [35], and stress [36] and the 2 bespoke questionnaires measured social anxiety symptoms, these results were unsurprising. Nevertheless, they were pronounced; according to Cohen guidelines for interpreting Cohen *d* [37], all these changes were large (Cohen *d*>0.8) except for the changes on the depression and anxiety subscales of DASS, which were medium (Cohen *d*>0.5) in magnitude. We must advise caution in interpreting these results, however, as they exclude data from noncompleters and there is no control condition with which to compare them.

The completion rate (102/369, 27.7%) was high in comparison with community completion rates of other self-guided CP interventions. Data from community users show lower completion rates than data from trials [16], and self-guided CP interventions have lower completion rates than therapist-assisted CP interventions [38]; it is, therefore, unsurprising that community adherence to self-guided CP is typically low. For example, the highest completion rate found in a recent review examining community usage of self-guided CP interventions for depression, anxiety, and mood enhancement was 19.5% [16], and the intervention that achieved this completion rate, CBT Psych, was actually an earlier version of Overcome Social Anxiety targeted toward stuttering populations. Other research has shown that for self-guided internet psychotherapy interventions, over 90% of users withdraw after only 2 sessions [39]. In comparison, less than one-third of users in our sample (112/369, 30.4%) withdrew before completing the prequestionnaires, module 1, and module 2, which together took users an average of 2 hours and 13 min. It should be noted, however, that there is some experimental evidence suggesting that adding a financial cost to ICBT interventions increases adherence [32]. Some proportion of Overcome Social Anxiety’s comparatively high adherence rate may, therefore, be attributable to its cost. Indeed, it would appear reasonable to expect that users who are willing to make financial sacrifices to access ICBT interventions are also more willing to sacrifice the time and effort necessary to complete those interventions, whereas those who are less committed may tend to opt for free ICBT instead.

Finally, user feedback was generally positive. For example, of the 35 completers who left feedback, 26 (26/35, 74%) left general praise and 18 (18/35, 51%) specifically stated that they had experienced a reduction in symptom severity. However, because feedback was solicited only at the end of the intervention, users who enjoyed and benefited from the intervention may be overrepresented, whereas users who did not enjoy or benefit from it may have tended to drop out before reaching the user feedback questions.

### Limitations and Future Research

There remain a number of important questions for future research to address. First, although it has been shown that a therapist’s assistance increases adherence to CCBT [38], therapist-assisted CP is not as scalable as self-guided CP. The further development of mechanisms to improve adherence to self-guided CP interventions remains an important avenue for future research. Second, it is a limitation of this study that we have no posttreatment data from noncompleters and therefore cannot report changes in their symptom severity following their partial completion of the intervention. Although past research shows that partial completion of ICBT interventions is beneficial [32], and some participants may indeed have ceased using the intervention after experiencing a satisfactory reduction in the severity of their symptoms, future research measuring symptoms periodically over the course of the treatment would be required to clarify the effects of partially completing Overcome Social Anxiety. Third, the intervention’s apparent success cannot currently be attributed to any particular elements of its design. Overcome Social Anxiety was created to address 5 limitations of other ICBT interventions, but it remains unclear which of these limitations are most crucial for designers of future ICBT interventions to address. Fourth, although Overcome Social Anxiety has now been evaluated as an intervention for university undergraduates [14] and members of its general user base, future research would be required to evaluate the intervention in a clinical setting or to compare its effectiveness with human-delivered CBT. On a related note, although high FNE scores among our participants indicate that many of them may have met diagnostic criteria for social anxiety disorder, our lack of diagnostic interviews leaves us unable to draw any conclusions about the intervention’s efficacy among those who did. Fifth, additional research exploring the predictors of program completion and symptom reduction could be useful in targeting the program toward those most likely to benefit from it or in establishing a screening mechanism to help prospective users decide whether it is an appropriate treatment for them. To this end, given our finding that completers had lower scores than noncompleters on the anxiety subscale of DASS, research examining that measure as a predictor of program outcomes could be considered. Sixth, the fact that the majority of users were men is interesting as a number of studies have shown that social anxiety is more prevalent among women [40]. Future research could explore the possibility that men value the privacy afforded by stand-alone ICBT programs more highly than women do. Finally, there is currently no data indicating whether and for what length of time users maintain reductions in symptom severity following their completion of the intervention.

### Conclusions

Overcome Social Anxiety was initially evaluated through a randomized controlled trial, which indicated that the intervention reduces the severity of social anxiety symptoms among university undergraduates [14]. Although our study’s lack of a control condition leaves us unable to draw causal inferences, we believe that it is reasonable to suppose that a considerable proportion of the pretreatment-to-posttreatment reduction in symptom severity may be attributable to the intervention, as this was a very large effect (Cohen *d*=1.76) and research shows that social anxiety tends to be persistent when it remains untreated [5]. Given this assumption, this study reinforces the findings of the initial trial [14] in 4 ways. First, it supports the finding that Overcome Social Anxiety is effective in reducing the severity of social anxiety symptoms. Second, its high external validity extends the initial trial’s results to indicate that the intervention is highly effective among community users who complete it. Third, it suggests that the intervention’s benefits are not limited to reducing the severity of social anxiety symptoms; the program appears to alleviate symptoms of depression, physiological symptoms of anxiety, and stress among its users as well. Finally, this study suggests that Overcome Social Anxiety has a high completion rate compared with other self-guided CP interventions. In summary, the results of this study converge with those of the initial trial’s, attesting to Overcome Social Anxiety’s effectiveness as a self-guided ICBT intervention and providing further indication that future interventions may be able to draw from elements of its design.

## Appendix

Multimedia Appendix 1 Screenshot of Overcome Social Anxiety.

## Abbreviations

CBT: cognitive behavioral therapy
CCBT: computerized cognitive behavioral therapy
CP: computer-aided psychotherapy
DASS: Depression Anxiety Stress Scales
FNE: Fear of Negative Evaluation Scale
ICBT: internet-delivered cognitive behavioral therapy
