# Supplementary figures and images for: Evaluating a Web-Based Social Anxiety Intervention Among Community Users: Analysis of Real-World Data
Source: J Med Internet Res. 2019 Jan 10;21(1):e11566. doi: 10.2196/11566 (PMC6329899; doi:10.2196/11566)

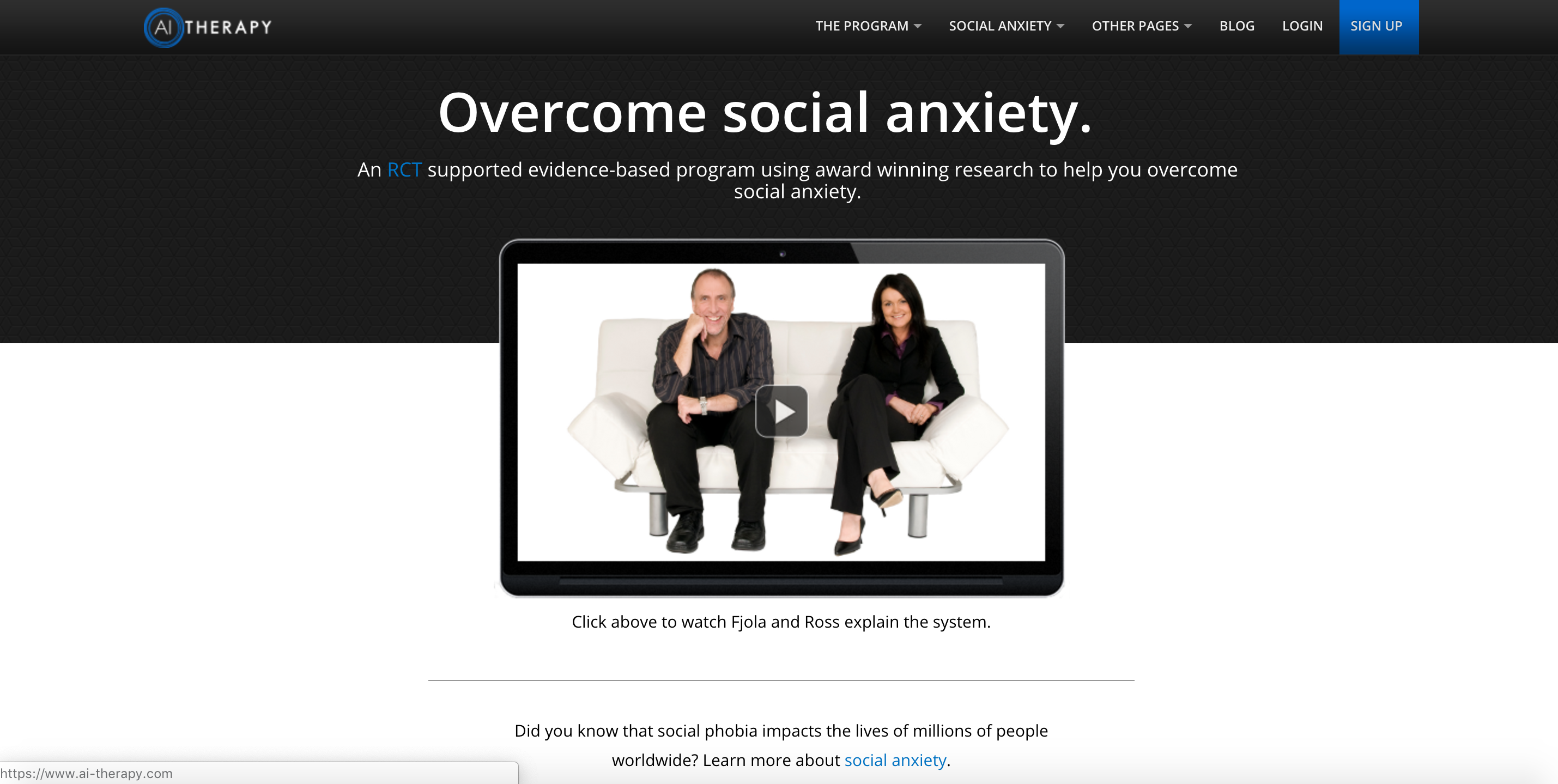

Supplement: Multimedia Appendix 1 [file jmir_v21i1e11566_app1.png]
